# Dynamic Mechanical Properties and Damage Constitutive Model of Frozen–Thawed Basalt Fiber-Reinforced Concrete Under Wide Strain Rate Range

**DOI:** 10.3390/ma18143337

**Published:** 2025-07-16

**Authors:** Wenbiao Liang, Siyi Wang, Xiao Lv, Yan Li

**Affiliations:** 1 School of Science, Chang’an University, Xi’an 710064, China; lwb23@chd.edu.cn; 2 School of Geological Engineering and Geodesy, Chang’an University, Xi’an 710064, China; liyanlwbdlp@chd.edu.cn; 3 Institute of Systems Engineering, China Academy of Engineering Physics, Mianyang 621900, China

**Keywords:** basalt fiber-reinforced concrete, dynamic mechanical properties, SHPB (Split-Hopkinson-Pressure-Bar), freeze–thaw action, damage constitutive model

## Abstract

To comprehensively investigate the compressive behavior of basalt fiber-reinforced concrete (BFRC) subjected to multiple freeze–thaw cycles, a series of quasi-static and dynamic compression tests were conducted on BFRC at various fiber volume fractions and a wide strain rate range of 1 × 10^−3^–420 s^−1^. The freeze–thaw deterioration characteristics of BFRC were analyzed from macro and micro perspectives. The influence of freeze–thaw degradation, strain rate effect, and fiber reinforcement effect on the mechanical performance of BFRC was investigated. It was found that when the fiber volume fraction was 0.2%, the fiber reinforcement performance of basalt fiber was optimal. By incorporating the damage factor of freeze–thaw cycles and the dynamic increase factor of strength into the Ottosen nonlinear elastic constitutive model, a dynamic constitutive model that considers the fiber content, strain rate enhancing effect, and freeze–thaw degradation influence was established.

## 1. Introduction

Due to seasonal changes and significant daily temperature differences and other factors, concrete structures such as dams and docks in the high-latitude regions of northern China and other similar climate regions abroad are often exposed to freeze–thaw cycles [1]. For instance, in the renowned Tianjin Port in Northern China, key structural components such as pile caps and prestressed piles often deteriorate to varying degrees due to the freeze–thaw process. The water in the internal pores of the concrete repeatedly freezes and melts, and the water–ice phase keeps changing, thereby reducing the durability of the components [2]. Furthermore, during the usage of hydraulic concrete buildings, they may be subjected to unpredictable dynamic loads such as earthquakes, tsunamis, and traffic accidents [3,4]. The dual effects of dynamic loads and freeze–thaw environments seriously affect the service life of concrete structures. Therefore, it is of great significance to study the physical and chemical properties and impact resistance of concrete materials under the action of freeze–thaw cycles.

Studies have shown that basalt fiber (BF) possesses excellent tensile strength [5], chemical stability [6], durability [7], and environmental sustainability [8], among other properties. In view of these excellent engineering mechanical properties of basalt fibers, some scholars have incorporated them into the concrete matrix to improve the engineering mechanical properties of plain concrete. For instance, Yang et al. [9] found through experimental research that increasing the volume content of BF could significantly enhance the static compressive strength of BFRC under ultra-low temperature cycling conditions. Zeng et al. [10] studied the quasi-static axial compression mechanical behavior of basalt fiber-reinforced lightweight aggregate concrete after freeze–thaw cycles. The results show that the incorporation of basalt fibers could significantly alleviate the stress and strain degradation caused by freeze–thaw cycles. Fu et al. [11] and Xie et al. [12], respectively, analyzed the dynamic mechanical properties of BFRC at strain rates of 20–140 s^−1^ and 80–220 s^−1^ based on experimental research. The results show that basalt fibers can enhance the dynamic compression performance and energy consumption characteristics of concrete. The above-mentioned research has laid a good foundation for comprehensively revealing the mechanical properties of BFRC under freeze–thaw cycles. However, during the entire service life of concrete structures, the strain rates that may occur under various loads are quite extensive [13]. Nevertheless, the current research on the changes in the mechanical properties of BFRC across a large strain rate range is relatively limited.

The constitutive model is the key to clarifying the mechanical properties of materials [14]. Reasonable constitutive parameters are the basis for the reliability design of fiber concrete engineering in the freeze–thaw environment. At present, the constitutive model of FRC is mainly achieved by modifying the constitutive model parameters of classical plain concrete [15,16,17]. For example, Xu et al. modified 64 material parameters in a KCC (Karagozian & Case Concrete) material model based on the experimental results, and a constitutive equation that can accurately predict the uniaxial compression mechanical behavior of UHP-FRC (Ultra-High-Performance Fiber-Reinforced Concrete) materials at a strain rate of 20 s^−1^ was obtained [18]. Based on the HJC (Holmquist-Johnson-Cook) model, Yang et al. introduced shear damage parameters and compaction damage parameters and proposed a constitutive model that can describe the strain hardening phenomenon of SFRC under impact loading. This constitutive model comprehensively considers the influences of multiple factors such as the strength of the concrete matrix, the shape of the fibers, and the volume content of the fibers [19]. Although a large number of studies have shown that the mechanical response of fiber concrete has a significant strain rate strengthening effect, freeze–thaw deterioration effect, and fiber toughening effect, the constitutive model considering the comprehensive characterization of multiple factors is very crucial for accurately predicting the mechanical behavior of the material. However, there are still relatively few constitutive models that comprehensively reflect the influence characteristics of multiple factors. Li et al. established the FRC constitutive model under a high strain rate in the freeze–thaw environment by introducing the dynamic strength increase factor (DIF) and the damage factor [20], providing a new idea for considering the influence of multiple factors in constructing the FRC constitutive model.

This study conducted freeze–thaw cycle tests on BFRC with different fiber dosages to analyze the deterioration mechanism of BFRC caused by freeze–thaw cycles. The strain rate range of the material was set at 1 × 10^−3^–100 s^−1^. Quasi-static and dynamic impact compression tests were conducted on the specimens after freeze–thaw cycle loading to study the influence laws of the number of freeze–thaw cycles, the strain rate of the material, and the fiber content on the damage characteristics and compressive dynamic mechanical properties of BFRC. Comprehensively considering the strain rate strengthening effect, freeze–thaw deterioration effect, and fiber toughening effect, a phenomenological damage constitutive model suitable for the multi-factor comprehensive characterization of BFRC is constructed.

## 2. Freeze–Thaw Cycle Tests and Compression Mechanics Tests

### 2.1. Specimen Preparation

**Figure 1:**
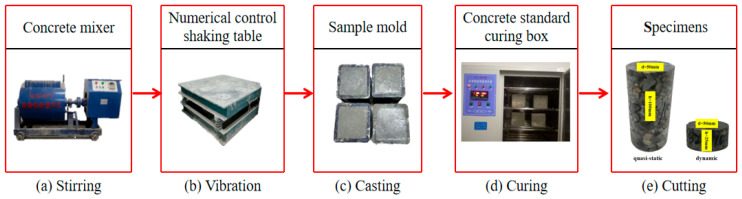
The instruments utilized in the preparation of BFRC.

In this study, C35 concrete was selected, and its mix proportion was elaborately designed strictly in accordance with the Chinese standard “Code for Design of Mix Proportions of Ordinary Concrete” (JGJ 55-2011) [21], as shown in Table 1. The basalt fiber (BF) used was produced by Shanghai Chenqi Chemical Technology Co., Ltd. (Shanghai, China) Its key parameters are shown in Table 2. According to previous research results [22], the test volume fractions of BF were set as 0%, 0.1%, 0.2%, 0.3%, and 0.4%, respectively. Figure 1 shows the preparation process of the sample. First, each component was mixed according to the mixing ratio shown in Table 1. To ensure the uniform mixing of basalt fiber with the concrete matrix, raw materials such as sand, crushed stone, and basalt fiber were dry-mixed first, and then cement materials, water, and water-reducing agent were added for the second round of mixing. After thoroughly mixing the concrete mixture, it was placed in a 150 mm × 150 mm × 150 mm test mold for vibration smoothing. Then, it was cured with air at a temperature of 25 °C and a relative humidity greater than 50% for 24 h. Finally, the specimens were transferred into the standardized curing box for curing for 28 days. After core drilling, cutting, and grinding, according to the Chinese standard “Standard Test Methods for Physical and Mechanical Properties of Concrete” (GB/T 50081-2019) [23], the quasi-static specimens were processed into cylinders with a diameter of 50 mm and a height of 100 mm, and the dynamic specimens were processed into cylinders with a diameter of 50 mm and a height of 25 mm.

### 2.2. Test Method

#### 2.2.1. Freeze–Thaw Cycle Tests

**Figure 2:**
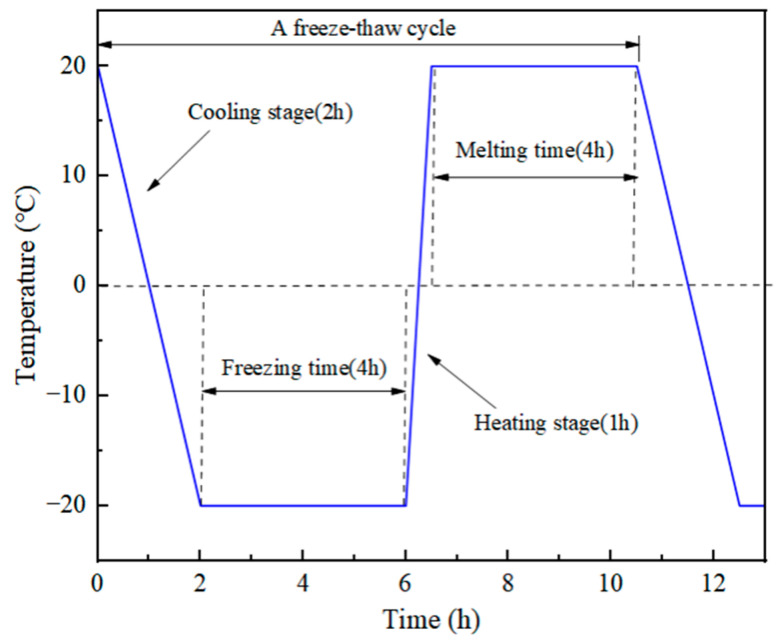
Time–temperature curve of a freeze–thaw cycle.

The freeze–thaw cycle test in this study was conducted in accordance with the Chinese standard “Standard Test Methods for Long-Term Properties and Durability of Ordinary Concrete” (GB/T 50082-2009) [24] in a high-precision low-temperature cycle test chamber (C3300A model with a temperature range of −30 to 100 °C). The minimum temperature of the test was set at −20 °C, and the maximum temperature was 20 °C. The number of freeze–thaw cycles was set at 0, 10, 30, and 50 cycles, respectively, which were counted as FT0, FT10, FT30, and FT50, respectively. It should be particularly noted that according to the pre-test results of this study, when the number of freeze–thaw cycles reaches 70 times, the freeze–thaw damage of the specimens is obvious and the integrity is poor, no longer meeting the requirements of mechanical tests. Therefore, the maximum number of freeze–thaw cycles in this study is 50 times. The temperature time history curve of one freeze–thaw cycle is shown in Figure 2. Firstly, the sample was gradually cooled from +20 °C to −20 °C for 120 min, with a cooling rate of approximately 0.33 °C/min. Then, it was continuously frozen at a constant temperature of −20 °C for 240 min. Subsequently, the sample was rapidly heated to 20 °C for 30 min, with a heating rate of approximately 1.33 °C/min. Finally, it was maintained at a temperature of 20 °C for 240 min. The total time of one freeze–thaw cycle amounts to 630 min.

#### 2.2.2. Quasi-Static and Dynamic Compression Tests

**Figure 3:**
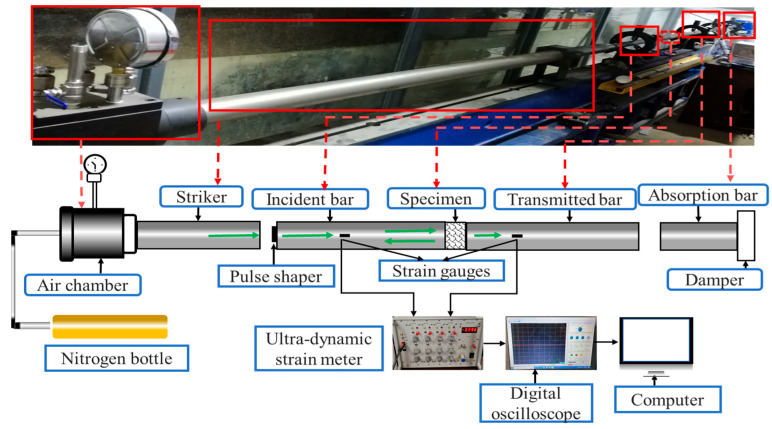
SHPB test device.

The quasi-static test was carried out on the microcomputer-controlled electro-hydraulic servo universal test equipment (WAW3000A, Hengle Xingke Instruments, Jinan, China), and the strain rate was set at 1 × 10^−3^ s^−1^. The dynamic pressure impact compression test was conducted on the 50-mm-diameter split-hopkinson-pressure-bar (SHPB) device in the Explosion and Impact Laboratory of Chang ‘an University (as shown in Figure 3), with the impact pressures set at 0.2 MPa, 0.4 MPa, and 0.6 MPa, respectively. The “three-wave method” [25] was adopted to analyze the collected dynamic test data.

In conclusion, in this study, a total of 80 working conditions were designed, including 5 different fiber dosages, 4 different freeze–thaw cycles, and 4 different loading rates (namely, 5 × 4 × 4 = 80). To ensure the reliability of the test results, three groups of samples were designed for each working condition for repeated tests, and the test results were taken as the average of the three groups of samples. Therefore, a total of 240 test samples were designed in this study.

## 3. Results and Discussion

### 3.1. Macro and Micro Damage Characteristics and Evolution Laws of BFRC Under Freeze–Thaw Cycling

**Figure 4:**
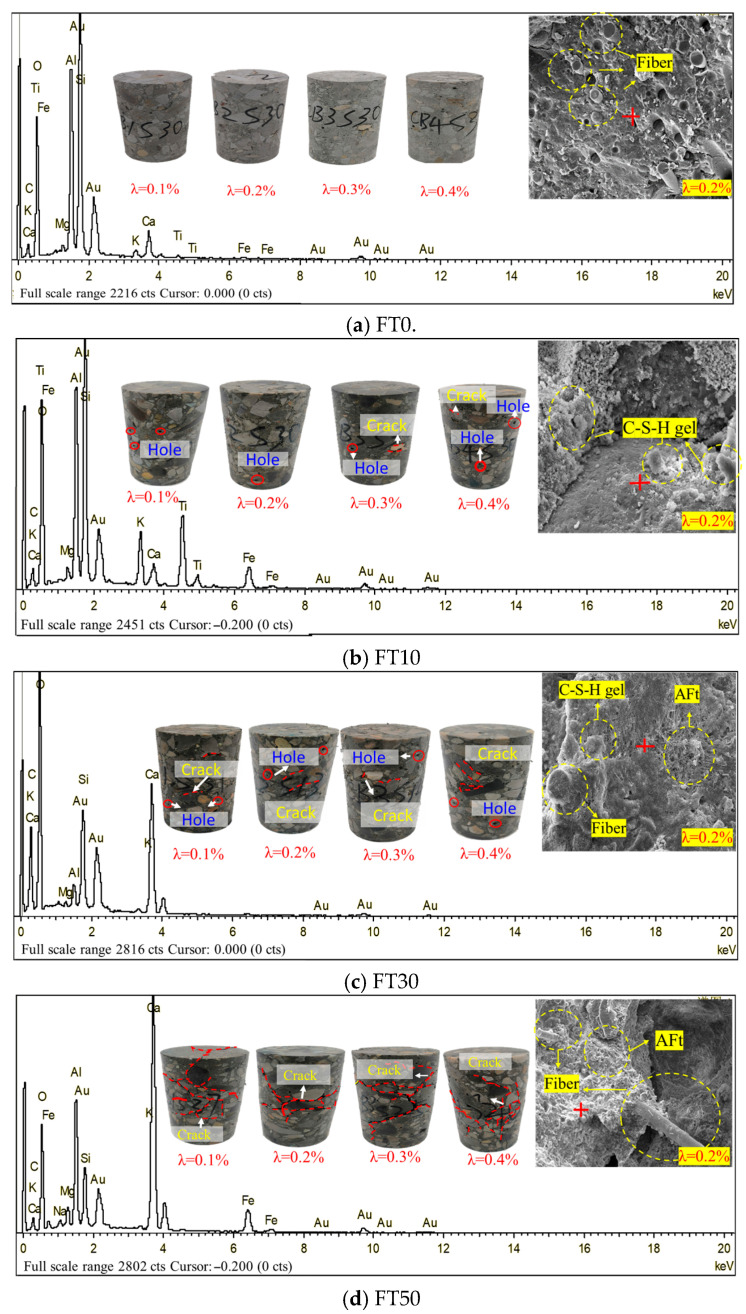
Macro and micro damage characteristics of BFRC (λ: fiber content) under freeze–thaw cycles.

In order to investigate the influence of freeze–thaw cycles on the damage of BFRC, the macro and micro morphology of BFRC samples under different working conditions were analyzed, as shown in Figure 4.

As shown in Figure 4, the freeze–thaw cycle has a significant deterioration effect on BFRC. Under the FT0 condition, the surface of the BFRC sample is smooth and there are sporadic natural voids. After 10 freeze–thaw cycles, fine cracks emerged at the aggregate–mortar interface of BFRC. With the further increase in the number of freeze–thaw cycles, the width of the microcracks increases and the cracks gradually aggregate. When the number of freeze–thaw cycles reaches 50, the main crack begins to penetrate, presenting a distribution feature of being dense in the middle and sparse at the upper and lower ends of the sample. Furthermore, by comparing the apparent deterioration characteristics of BFRC specimens with different fiber contents, it can be seen that when the fiber content is 0.2%, the freeze–thaw damage cracks of the specimens are the least, that is, the performance of resisting freeze–thaw deterioration is the best.

It can also be seen from Figure 4 that the microstructure of BFRC is composed of four phases: the cement slurry phase, the aggregate phase, the fiber phase, and the interface transition zone. With the increase in the number of freeze–thaw cycles, the characteristics of the microstructure have changed significantly. Under the FT0 condition, except for the initial micro-pores, the microstructure of BFRC is basically dense. When the freeze–thaw cycle is carried out 10 times, the water in the internal pores of BFRC repeatedly freezes and melts, and the water–ice phase keeps changing. On the one hand, when cement comes into contact with water, it undergoes hydration and hydrolysis reactions, forming slightly expanded calcium silicate hydrate (C-S-H) gel. This basic hydration reaction can last for months or even years [26]. On the other hand, the expansion stress of the ice phase generated by freeze–thaw leads to the initiation and propagation of fine cracks, disrupts the network structure of C-S-H gel, and accelerates pore connectivity and the precipitation of ettringite (AFt) crystals. Basalt fibers themselves do not participate in hydration reactions, but their physical distribution can promote uniform water distribution by blocking aggregate sedimentation, thereby optimizing the hydration environment. Moreover, through the “bridging” effect, they form a three-dimensional grid structure within the BFRC, effectively hindering physical damage caused by freeze–thaw cycles. These collectively lead to the evolution and development of the pore system in the microstructure of BFRC. When the number of freeze–thaw cycles reaches 30, the calcium silicate hydrate (C-S-H) gel continues to grow, and slender needle-like crystals gradually emerge and form slightly expanded clumps with the C-S-H gel. An energy spectrum analysis indicates that these crystals are mainly composed of Ca, C, O, Si, and Al elements, possibly ettringite (AFt), along with a small amount of calcium aluminate and Ca(OH)_2_ crystals. When the number of freeze–thaw cycles reaches 50, AFt continues to increase, and microcracks at the cement paste phase and the interface transition zone further develop. The change mechanism of the internal microstructure of BFRC is the essential driving factor for the apparent deterioration of its freeze–thaw damage.

**Figure 5:**
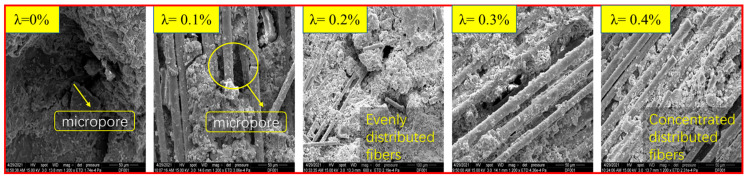
Microscopic images of BFRC (λ: fiber content) with different fiber contents under 0 freeze–thaw cycles.

Figure 5 shows the microscopic morphological characteristics of BFRC under different fiber dosages.

As can be seen from Figure 5, the inherent pore structure of plain concrete is clearly visible. When the volume content of fibers is 0.1%, the fibers can be well integrated with the concrete matrix. However, due to the insufficient content of fibers, there are still a certain number of pores in the concrete. When the fiber content increases to 0.2%, the fibers form a denser three-dimensional grid microstructure with the concrete matrix. When the volume content of fibers continues to increase to 0.3% and 0.4%, the increase in volume content leads to the “agglomeration” of fibers within the concrete, resulting in an uneven distribution within the concrete and instead becoming a weak point in the internal structure of BFRC. The dispersion effect and uniformity of fibers in concrete undoubtedly directly affect the macroscopic mechanical properties of basalt fiber concrete. The surface of basalt fibers is smooth and lacks polar hydrophilic groups such as amino and carboxyl groups. This results in a weak interaction between the fibers and water, and they are prone to forming “agglomerations” in the cement paste. These factors lead to the difficulty of dispersion of basalt fibers in the concrete matrix and poor dispersion stability, thereby affecting the uniformity of basalt fiber concrete; this phenomenon becomes more pronounced when the volume fraction of fibers is large.

### 3.2. Dynamic Failure Patterns and Block Size Distributions

**Figure 6:**
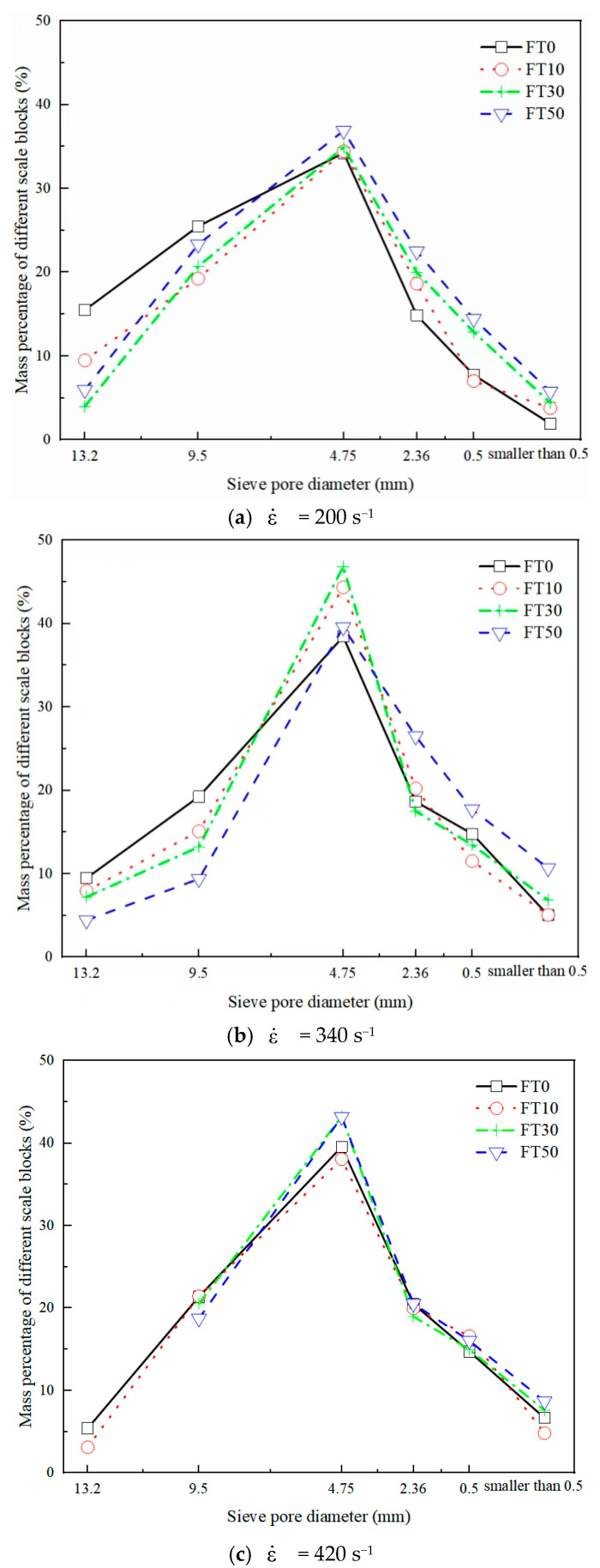
Mass–size distribution diagram and failure morphology of fragments.

After calculation, under the impact pressures of 0.2 MPa, 0.4 MPa, and 0.6 MPa, the strain rates of BFRC are 200 s^−1^, 340 s^−1^, and 420 s^−1^ respectively. In order to analyze the failure mode and size distribution characteristics of crushed BFRC blocks under different working conditions, this study took the working condition with a fiber volume content of 0.2% as an example. The standard vibrating screen (ZBSX−92A, Leiyun Experimental Instrument Manufacturing, Shanghai, China) was used to conduct screening tests on the impacted crushed blocks, and the distribution law of BFRC blocks obtained is shown in Figure 6.

As shown in Figure 6, the proportion of crushed blocks with a particle size range of 4.75 mm to 9.5 mm is the largest, accounting for 34.46% to 46.85% in total. The more freeze–thaw cycles there are or the higher the strain rate, the lower the mass in the large particle size range and the greater the mass in the small particle size range. This indicates that both freeze–thaw cycles and impact rates will intensify the failure degree of BFRC specimens. For example, under the working condition of a strain rate of 200 s^−1^, when the number of freeze–thaw cycles increased from 0 to 30 times, the mass percentage of particles larger than 13.2 mm decreased significantly from 15.2% to 4.03%, while the mass percentage of small particles (≤0.5 mm) gradually increased from 1.97% to 4.45%. Under the FT50 working condition, as the strain rate increased from 200 s^−1^ to 420 s^−1^, the mass percentage of particles larger than 13.2 mm decreased from 6% to 0%, while the mass percentage of small particles (≤0.5 mm) increased from 5.78% to 8.73%. The main reason for this phenomenon is that under the conditions of a lower number of freeze–thaw cycles or a lower strain rate, the energy absorbed by the sample is mainly dissipated through the crack propagation in the transition zone of the aggregate–slurry interface. However, under the effect of multiple freeze–thaw cycles, the freeze–thaw cracks in the concrete increase, splitting the specimens into numerous tiny blocks. At a high strain rate, the impact energy absorbed by the material cannot expand along the path with the least resistance in a short period of time. Instead, more fine cracks are generated, resulting in more thorough fracture of the specimen.

### 3.3. Influence of Freeze–Thaw Cycles and Strain Rates on Mechanical Properties

**Figure 7:**
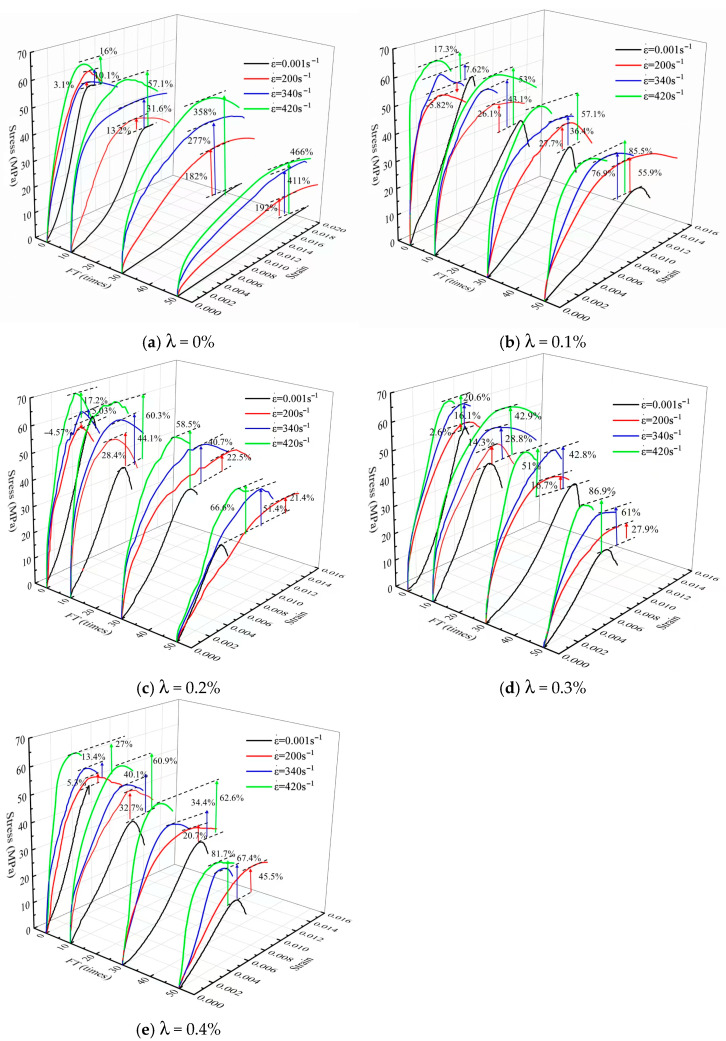
Stress–strain curves of BFRC under different freeze–thaw cycles.

The stress–strain curves of BFRC under different freeze–thaw cycles in a large strain rate range of 0.001 s^−1^–420 s^−1^ are displayed in Figure 7.

It can be seen from Figure 7 that the mechanical properties of BFRC have a significant strain rate strengthening effect. That is, with the increase in the strain rate, both the elastic modulus and the peak stress of BFRC increase. Furthermore, Figure 7 also indicates that the static and dynamic mechanical properties of BFRC have a significant freeze–thaw degradation effect. That is, with the increase in the number of freeze–thaw cycles, both the dynamic and quasi-static strengths of BFRC steadily decrease. Moreover, the gap between the static and dynamic compressive strengths of BFRC continuously expands with the increase in the number of freeze–thaw cycles, which is specifically manifested as follows: under the FT0 working condition, the difference between the dynamic strength and the quasi-static strength of BFRC is 2.6% to 27%. Under the FT50 working condition, the difference between the two increases from 27.9% to 466%.

### 3.4. Influence of Basalt Fiber on Compressive Strength

**Figure 8:**
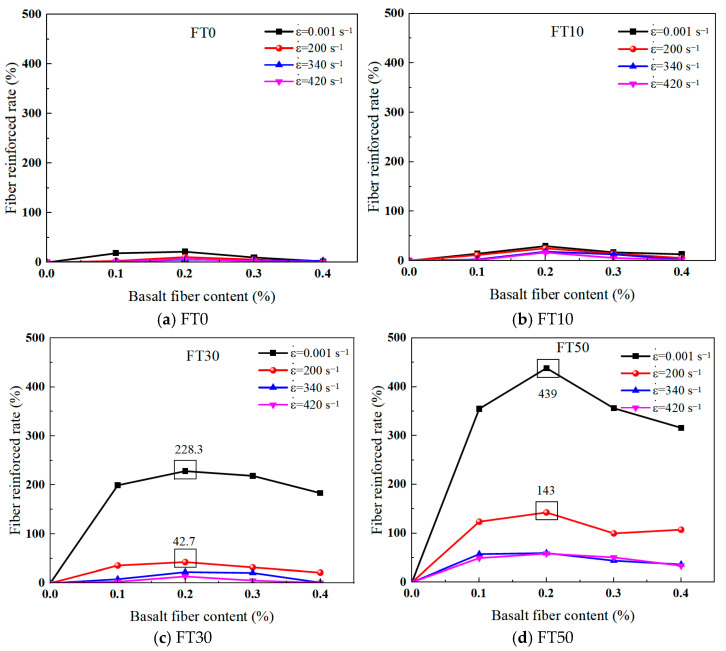
Variation curves of fiber reinforcement rate of BFRC under different strain rates.

In order to quantitatively analyze the influence of fiber content on the compressive strength of BFRC, the fiber reinforcement rate (FRR) is defined, as shown in Equation (1). Combined with the experimental data, the FRR of BFRC at different strain rates ($\beta_{f}^{n}$) was calculated according to Equation (1), and the results are shown in Figure 8.

(1) $$\beta_{f}^{n}=\frac{f_{\lambda }^{n}-f_{0}^{n}}{f_{0}^{n}}\times 100\%$$

where λ is the basalt fiber content, and $f_{\lambda }^{n}$ and $f_{0}^{n}$ are the strength value of BFRC and plain concrete after *n* freeze–thaw cycles, respectively.

As can be seen from Figure 8, the compressive strength of BFRC first increases and then decreases with the increase in the volume content of basalt fibers. When the volume content of fibers is 0.2%, the compressive strength of BFRC is the highest, and the more freeze–thaw cycles there are, the more significant the fiber reinforcing effect becomes. This experimental result is consistent with the experimental phenomenon shown in Figure 4 of Section 3.1 and with reference [27]. The research results are consistent.

It can be seen from Figure 8, the fiber reinforcement effect also shows dependence on the strain rate of the material, that is, the lower the strain rate, the more significant the fiber reinforcement effect. For example, under the FT50 working condition, the compressive strength of BFRC with a fiber volume content of 0.2% increased by 439% when the strain rate was 0.001 s^−1^ and increased by 143% when the strain rate was 200 s^−1^. The main reason for the dependence of the fiber-reinforced effect on the strain rate of the material might be that when the strain rate is low, the micro-cracks in BFRC have sufficient time to expand and connect with each other. During this process, the fibers fully play their interlinking role, effectively preventing the rapid expansion of cracks and thereby enhancing the compressive strength. However, under the condition of a high strain rate, the BFRC specimens drove the initiation of new cracks by absorbing energy in an extremely short time, and the fiber reinforcement effect was relatively insignificant.

## 4. Damage Constitutive Model of BFRC Considering Both Freeze–ThawFreeze-Thaw Action and Strain Rate Effect

### 4.1. Ottosen Nonlinear Elastic Static Constitutive Model

The Ottosen constitutive model is a classical constitutive model that describes the nonlinear elastic mechanical behavior of concrete-like materials. Its static stress–strain curve is shown in Equation (2) [28].

(2) $$\sigma_{s}=f_{c,s}\frac{A_{1}\frac{\varepsilon }{\varepsilon_{p,s}}+(A_{2}-1){\left(\frac{\varepsilon }{\varepsilon_{p,s}}\right)}^{2}}{1+(A_{1}-2)\frac{\varepsilon }{\varepsilon_{p,s}}+A_{2}{\left(\frac{\varepsilon }{\varepsilon_{p,s}}\right)}^{2}}$$

where $\sigma_{s}$ is the static pressure, $f_{c,s}$ is the static compressive strength, ε is the strain, $\varepsilon_{p,s}$ is the peak strain, *A_1_* and *A_2_* are both parametric models, where: $A_{1}$ is the ratio of the initial elastic modulus $\left(E_{0}\right)$ to the secant modulus $\left(E_{c}\right)$ at the peak stress, *A*_2_ is calculated based on the test results of the specific material.

### 4.2. Freeze–Thaw Damage Factor

In this study, the freeze–thaw damage factor was introduced to characterize the damage caused by freeze–thaw cycles to the materials. Studies have shown [29] that the relationship between the freeze–thaw cycle factor and the number of freeze–thaw cycles can be described by the Weibull distribution, as shown in Equation (3):

(3) $$D_{c}=1-\frac{\sigma_{sn}}{\sigma_{s0}}=1-p\cdot e^{-qn}$$

where $D_{c}$ is the freeze–thaw damage factor. $\sigma_{s0}$ and $\sigma_{sn}$ represent the quasi-static compressive strength of BFRC after zero and *n* freeze–thaw cycles, respectively. *p* and *q* are fitting parameters, and their values are illustrated in Table 3.

**Figure 9:**
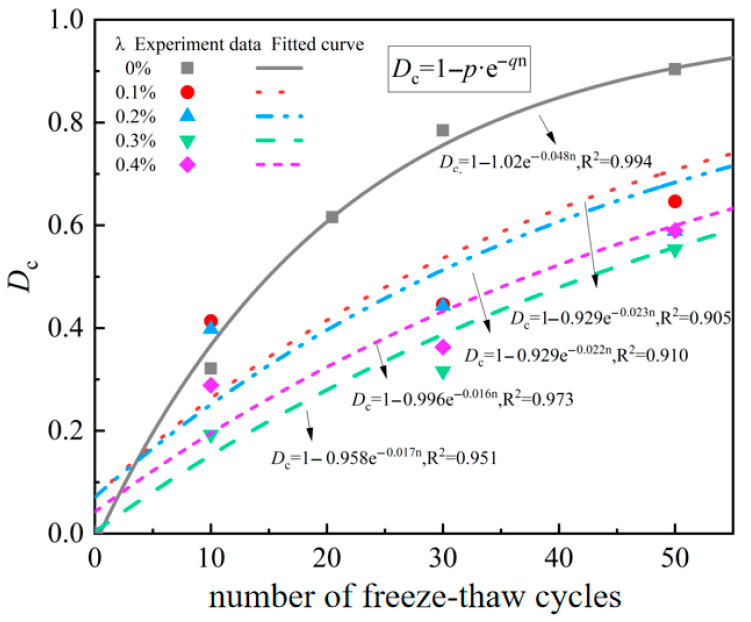
Relationship between $D_{c}$ and freeze–thaw cycles.

It can be intuitively seen from Figure 9 that under different working conditions, the freeze–thaw damage factor of BFRC increases exponentially with the increase in the number of freeze–thaw cycles, and the freeze–thaw damage factor of plain concrete is significantly greater than that of fiber concrete.

Furthermore, it can be seen from Formula (3) and Figure 9 that when *n* = 0, the obtained $D_{c}$ value is 1 − *p*, which is reflected as the longitudinal intercept of each curve, indicating the initial degree of freeze–thaw damage. The lower the 1 − *p* value is, the smaller the initial damage will be. The parameter q mainly reflects the deterioration rate of BFRC. The higher the *q* value is, the faster the freeze–thaw deterioration rate of BFRC is. It can be seen from the parameter values in Table 3 that the freeze–thaw deterioration rate of plain concrete is the highest, and its q value is 0.048. The minimum freeze–thaw deterioration rate q value of BFRC is 0.016. This indicates that basalt fibers can effectively enhance the frost resistance of concrete and reduce the accumulation degree of freeze–thaw damage. This result is consistent with the test phenomenon in Figure 4 of Section 3.1 mentioned above.

### 4.3. Dynamic Increase Factor of Compressive Strength

**Figure 10:**
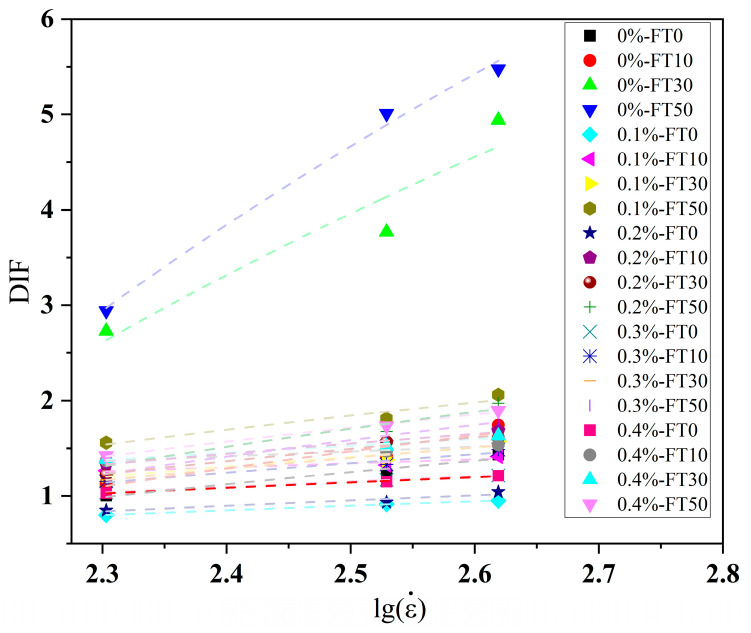
*DIF* values under different working conditions.

The dynamic increase factor (*DIF*) of compressive strength is defined as the ratio of dynamic compressive strength to quasi-static compressive strength. The *DIF* values under different freeze–thaw cycle periods are shown in Figure 10. By fitting the experimental data, it was found that there was a linear relationship between *DIF* and the logarithm of the strain rate, as shown in Equation (4). This research conclusion is consistent with reference [30].

(4) $$DIF=\frac{\sigma_{dn}}{\sigma_{sn}}=A\mathrm{lg}\dot{\varepsilon }+B$$

where $\sigma_{dn}$ and $\sigma_{sn}$ are the dynamic and quasi-static compressive strengths after *n* freeze–thaw cycles, respectively, and *A* and *B* are the fitting parameters related to the number of freeze–thaw cycles and fiber content.

As can be seen from Figure 10, the value of the dynamic strength growth factor (*DIF*) is linearly and positively correlated with the strain rate. Moreover, compared with plain concrete, the incorporation of basalt fibers will reduce the sensitivity of concrete to the strain rate. This is mainly because the fibers form a three-dimensional network structure in the concrete matrix, which disperses local stress through bridging and reduces strain concentration in the matrix. Meanwhile, BFRC absorbs energy through pull-out and the deformation of fibers during the force application process, enhancing the toughness characteristics and weakening the dependence on the strain rate. For instance, under the FT30 and FT50 conditions, the DIF values of plain concrete samples fluctuated between 2.5 and 5.0, while those of the BFRC samples fluctuated between 1.0 and 2.0.

### 4.4. A Constitutive Model of Freeze–Thaw Damage Considering the Strain Rate Effect and the Fiber Toughening Effect

In order to construct a constitutive model of BFRC that comprehensively considers the freeze–thaw damage deterioration effect and the strain rate strengthening effect, based on the Ottosen nonlinear constitutive model, the freeze–thaw damage factor and the dynamic strength growth factor are introduced, as shown in Equation (5).

(5) $$\sigma =f(D,DIF,\sigma_{s})=(1-D)\cdot DIF\cdot \sigma_{s},$$

where σ is the stress. Substituting Equations (2)–(4) into Equation (5) yields the following result:

(6) $$\sigma =f(D,DIF,\sigma_{s})=(pe^{-qn})(A\mathrm{lg}\dot{\varepsilon }+B)\left[f_{c,s}\frac{A_{1}\frac{\varepsilon }{\varepsilon_{p,s}}+(A_{2}-1){\left(\frac{\varepsilon }{\varepsilon_{p,s}}\right)}^{2}}{1+(A_{1}-2)\frac{\varepsilon }{\varepsilon_{p,s}}+A_{2}{\left(\frac{\varepsilon }{\varepsilon_{p,s}}\right)}^{2}}\right]$$

By replacing $\varepsilon_{p,s}$ in Equation (6) with $\varepsilon_{p,d}$, a dynamic constitutive model of frozen–thawed BFRC was obtained:

(7) $$\sigma =f(D,DIF,\sigma_{s})=(pe^{-qn})(A\mathrm{lg}\dot{\varepsilon }+B)\left[f_{c,s}\frac{A_{1}\frac{\varepsilon }{\varepsilon_{p,d}}+(A_{2}-1){\left(\frac{\varepsilon }{\varepsilon_{p,d}}\right)}^{2}}{1+(A_{1}-2)\frac{\varepsilon }{\varepsilon_{p,d}}+A_{2}{\left(\frac{\varepsilon }{\varepsilon_{p,d}}\right)}^{2}}\right]$$

where *p, q, A, B*, $A_{1}$, and $A_{2}$ are parameters.

### 4.5. Parameter Determination and Model Verification

In Equation (7), the values of parameters *p* and *q* are determined according to the method given in Section 4.2. A regression analysis was conducted based on the test data under strain rate conditions of 200 s^−1^ and 340 s^−1^, and the values of *A*, *B*, $A_{1}$*,* and $A_{2}$ were obtained as shown in Table 4.

**Figure 11:**
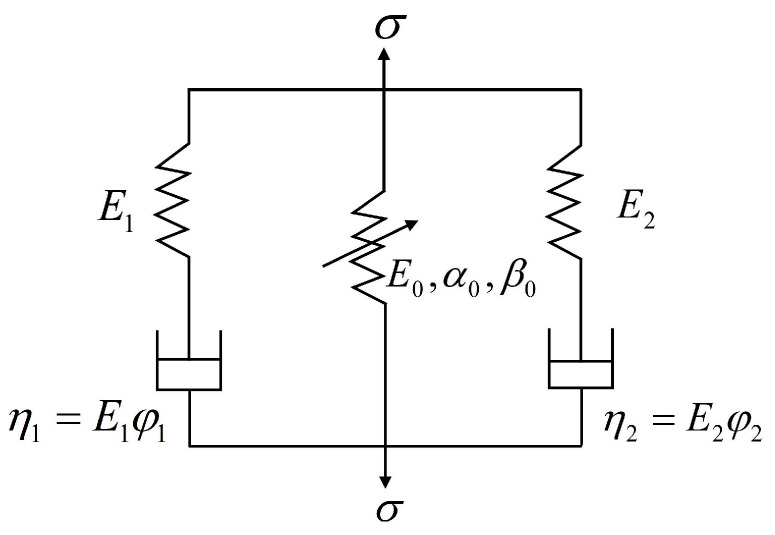
ZWT constitutive model.

The Zhu–Wang–Tang (ZWT) model is a nonlinear viscoelastic constitutive model applicable to strain rates ranging from 10^−4^ s^−1^ to 10^3^ s^−1^ [31]. The ZWT model is shown in Figure 11. This model consists of a nonlinear elastic element in parallel with two Maxwell elements. The first Maxwell element (with parameters *E*_1_ and $\varphi_{1}$) describes the viscoelastic response under quasi-static and low-strain-rate conditions, while the second Maxwell element (with parameters *E*_2_, $\varphi_{2}$,) describes the viscoelastic response under high-strain-rate conditions.

The expression of the ZWT model is as follows:

(8) $$\sigma =E_{0}\varepsilon +\alpha \varepsilon^{2}+\beta \varepsilon^{2}+E_{1}\int_{0}^{t}\varepsilon (\tau )\exp(-\frac{t-\tau }{\varphi_{1}})d\tau +E_{2}\int_{0}^{t}\varepsilon (\tau )\exp(-\frac{t-\tau }{\varphi_{2}})d\tau$$

In Equation (8), *E*_0_, *E*_1_, $\varphi_{1}$, *E*_2_, and $\varphi_{2}$ are material constants; *E*_0_, α, and β are elastic constants; *E*_1_ and *E*_2_ are linear elastic moduli; and $\varphi_{1}$ and $\varphi_{2}$ are relaxation times.

In the SHPB test of concrete materials, the deformation of the specimens is very small, and the stress–strain curves in the quasi-static test are approximately linear. Therefore, the first nonlinear elastic part in the above equation can be regarded as linear elastic, and only its first term can be taken. The SHPB test can be approximately considered as a constant strain rate loading process. Thus, the expression of the ZWT model considering damage can be written as

(9) $$\sigma =\exp[-\frac{\varepsilon^{m}}{\alpha }]\left[E_{0}\varepsilon +E_{1}\varphi_{1}\dot{\varepsilon }(1-e^{-\frac{\varepsilon }{\dot{\varepsilon }\varphi_{1}}})+E_{2}\varphi_{2}\dot{\varepsilon }(1-e^{-\frac{\varepsilon }{\dot{\varepsilon }\varphi_{2}}})\right]$$

**Figure 12:**
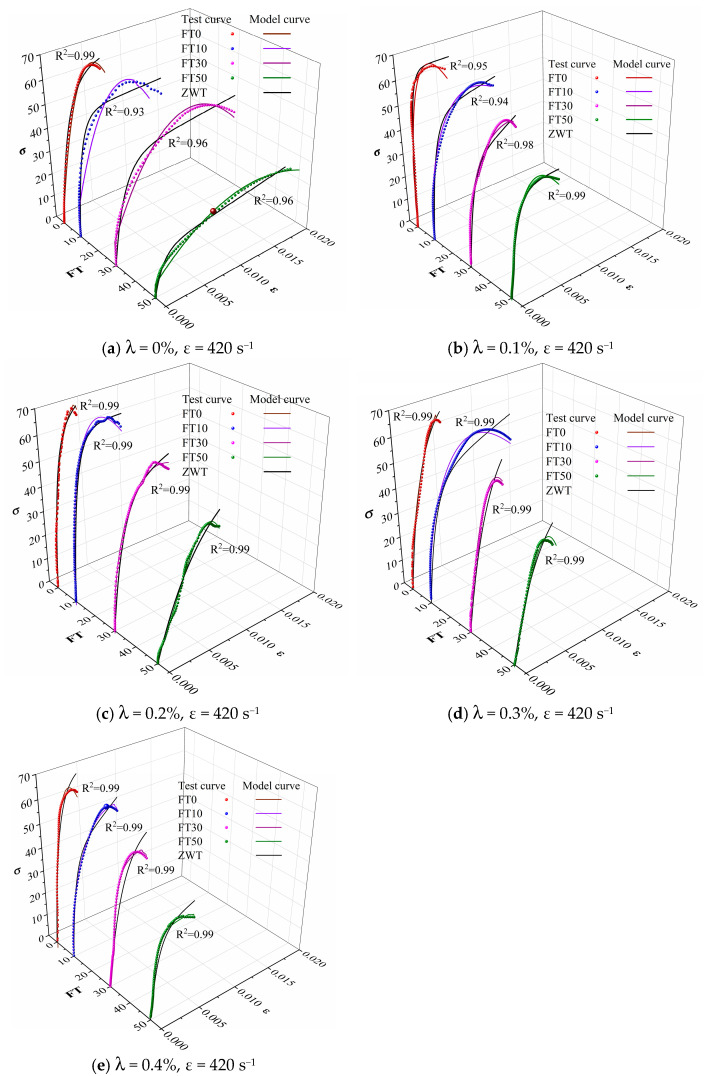
A comparison chart of theoretical calculation values and experimental values.

A comparison among the theoretical calculation values of the constitutive model in this study, the calculation values of the ZWT model, and the experimental values are shown in Figure 12.

As shown in Figure 12, the stress–strain curves calculated based on the dynamic damage constitutive model (Equation (7)) constructed in this study are in good agreement with the experimental data, and the goodness of fit R^2^ values (as shown in Figure 12) are all greater than 0.93. Meanwhile, the dynamic damage constitutive model proposed in this paper has fewer parameters and a higher goodness-of-fit R^2^ compared with the ZWT model. Therefore, this constitutive model well characterizes the dynamic mechanical response behavior of BFRC within the range of large strain rates under the influence of freeze–thaw cycles and can provide a reference value for the structural design of basalt fiber-reinforced concrete in cold regions.

## 5. Conclusions

(1) The freeze–thaw cycle has a significant deterioration effect on BFRC, and the deterioration mechanism can be summarized as follows: The water in the internal pores of BFRC repeatedly freezes and melts, and the water–ice phase keeps changing. During this process, the expansion stress of the ice phase causes the initiation and development of fine cracks. Meanwhile, the increasing water phase further hydrates the cement, forming slightly expanded calcium silicate hydrate gel, which further develops the pore system in the microstructure of BFRC. The change mechanism of the internal microstructure of BFRC is the essential driving factor for its freeze–thaw damage deterioration.
(2) BFRC has obvious strain rate strengthening and fiber toughening effects. With the increase in the strain rate, both the elastic modulus and peak stress of BFRC increase. The compressive strength of BFRC first increases and then decreases with the increase in the volume content of basalt fibers. When the volume content of the fibers is 0.2%, the compressive strength of BFRC is the highest. Moreover, due to the interlinking effect and energy absorption and dissipation of the fibers, the toughening effect of the fibers also shows a dependence on the strain rate of the material. That is, the lower the strain rate, the more significant the toughening effect of the fibers.
(3) Based on the Ottosen nonlinear elastic static constitutive model, a constitutive model that comprehensively considers the freeze–thaw damage deterioration effect, strain rate strengthening effect, and fiber toughening effect was constructed. This model can well describe the dynamic mechanical response behavior of BFRC in the range of large strain rates in the freeze–thaw cycle environment and can provide a reference for the structural design of BFRC in cold regions.

## Figures and Tables

**Table 1:** Proportions of various ingredients in the prepared C35 concrete.

Fiber Content (%)	Mix Ratio of Each Component of Concrete (kg·m^−3^)						
	Cement	Sand	Gravel	Water	Water Reducer	Basalt Fiber	Fly Ash
0.0	286.4	621.8	1262.6	157.5	5.4	0	71.6
0.1	286.4	621.8	1262.6	157.5	5.4	2.702	71.6
0.2	286.4	621.8	1262.6	157.5	5.4	5.409	71.6
0.3	286.4	621.8	1262.6	157.5	5.4	8.121	71.6
0.4	286.4	621.8	1262.6	157.5	5.4	10.839	71.6

**Table 2:** Basic properties of basalt fiber.

Fiber Type	Diameter (μm)	Length (mm)	Density (kg·m^−3^)	Elastic Modulus (GPa)	Melting Point (℃)	Ultimate Elongation (%)	Tensile Strength (MPa)	Basalt Fiber Picture
BF	17.4	12	2699	93–110	750	3.10	4150–4800	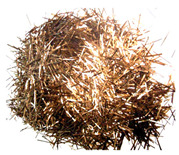

**Table 3:** Freeze–thaw damage parameters and fitting variance of the freeze–thaw damage evolution equation.

BF Content (%)	Freeze–Thaw Damage Parameters		Fitting Variance
	*p*	*q*	*R* ^2^
0	1.020	0.048	0.994
0.1	0.929	0.023	0.905
0.2	0.929	0.022	0.910
0.3	0.996	0.016	0.973
0.4	0.958	0.017	0.951

**Table 4:** Values of parameters *A*, *B*, $A_{1}$, and $A_{2}$ under different freeze–thaw cycles.

Freeze–Thaw Cycles	*A*	*B*	*A*_1_	*A*_2_
0	−0.434 + 9.116λ − 17.25λ^2^	2.116 − 22.71λ + 44.6λ^2^	1.245	0.316
10	−0.154 + 8.816λ − 17.25λ^2^	1.586 − 22.22λ + 44.6λ^2^	0.792	0.415
30	0.406 + 8.216λ − 17.25λ^2^	0.526 − 21.24λ + 44.6λ^2^	0.981	0.125
50	0.966 + 7.616λ − 17.25λ^2^	− 0.534 − 20.26λ + 44.6λ^2^	1.284	0.768

## Data Availability

The original contributions presented in this study are included in the article. Further inquiries can be directed to the corresponding author.
